# Improved electrochemical properties of morphology-controlled titania/titanate nanostructures prepared by in-situ hydrothermal surface modification of self-source Ti substrate for high-performance supercapacitors

**DOI:** 10.1038/s41598-017-11346-2

**Published:** 2017-10-16

**Authors:** Arghya Narayan Banerjee, V. C. Anitha, Sang W. Joo

**Affiliations:** 1 School of Mechanical Engineering, Yeungnam University, Gyeongsan, 712-749 Republic of Korea; 2 Center of Materials and Nanotechnologies, Faculty of Chemical Technology, University of Pardubice, Nam. Cs. Legii 565, 53002 Pardubice, Czech Republic

## Abstract

Ti substrate surface is modified into two-dimensional (2D) TiO_2_ nanoplatelet or one-dimensional (1D) nanorod/nanofiber (or a mixture of both) structure in a controlled manner via a simple KOH-based hydrothermal technique. Depending on the KOH concentration, different types of TiO_2_ nanostructures (2D platelets, 1D nanorods/nanofibers and a 2D+1D mixed sample) are fabricated directly onto the Ti substrate surface. The novelty of this technique is the *in-situ* modification of the self-source Ti surface into titania nanostructures, and its direct use as the electrochemical microelectrode without any modifications. This leads to considerable improvement in the interfacial properties between metallic Ti and semiconducting TiO_2_. Since interfacial states/defects have profound effect on charge transport properties of electronic/electrochemical devices, therefore this near-defect-free interfacial property of Ti-TiO_2_ microelectrode has shown high supercapacitive performances for superior charge-storage devices. Additionally, by hydrothermally tuning the morphology of titania nanostructures, the electrochemical properties of the electrodes are also tuned. A Ti-TiO_2_ electrode comprising of a mixture of 2D-platelet+1D-nanorod structure reveals very high specific capacitance values (~7.4 mF.cm^−2^) due to the unique mixed morphology which manifests higher active sites (hence, higher utilization of the active materials) in terms of greater roughness at the 2D-platelet structures and higher surface-to-volume-ratio in the 1D-nanorod structures.

## Introduction

TiO_2_ is a versatile and technologically important material that has a wide variety of applications, from paint to sunscreen to food coloring to photocatalyst, hydrogen production, storage medium, sensors, solar cells, transparent conductors, field emitters and various biological/health-related applications^1–6^. Low-dimensional (such as 0D, 1D, 2D) titania nanostructures exhibit some interesting properties like high surface-to-volume ratio, well-defined surface structure and geometry, low radii of curvature, better electrical, thermal and chemical stability etc., due to which TiO_2_ nanomaterials become important candidates for various surface-related/interfacial applications like electrochemical capacitors, photocatalysis, sensors and energy storage devices^7,8^. Amongst these applications, nanostructured TiO_2_, as a supercapacitor electrode material, has been explored in recent years for possible charge storage devices^9,10^. This is because, TiO_2_-based supercapacitors have exhibited promising energy storage capability by virtue of their good power/energy density, long life cycle, quick charging-discharging properties, high reversibility, wide operating temperature range and low cost per cycle^11,12^.

Generally, the charge storage mechanism in metal-oxide - based supercapacitors follows the faradaic reactions (reversible redox reactions) at the electrode surface (which are called faradaic supercapacitors or pseudocapacitors)^13–15^. But, by adding some carbon-based nanomaterials (which are called non-faradaic supercapacitors that are dominated by the electrostatic charge diffusion and accumulation at the electrode-electrolyte interface via an electric double-layer capacitor formation^16,17^) into the metal oxide matrix, capacitive performance can be improved considerably via the formation of hybrid supercapacitors^11,18,19^.

As far as the electrochemical performance of TiO_2_ is concerned, in general, it showed electrochemical double layer capacitance (non-faradaic) with considerably lower values of specific capacitances against the conventional carbonaceous non-faradaic capacitors^20^. This is mainly because of the semiconducting nature of titania that limits the conductivity and the presence of interfacial states/defects between titania - metal electrodes that prevents the fast charge transfer for better electrochemical properties^21^. To enhance the specific capacitance, various surface states are introduced onto the titania surface (via nanostructuring, phase transformation, oxygen vacancy creation, other non-metal/metal/metal-oxide incorporation etc.^21–24^), due to which faradaic reactions at the surfaces are manifested, leading to the introduction/enhancement of the pseudocapacitance, and hence, an overall increment in the specific capacitances.

Additionally, by enhancing the specific surface area of the electrodes (via controlling the morphology of the surface into porous and/or 0D/1D/2D nanostructures), greater utilization of the active materials is manifested, leading to the considerable enhancement of the specific capacitance of the nanomaterials^25,26^. Therefore, proper understanding of the surface and interfacial properties of the TiO_2_-based nanomaterials and the effective control of the surface morphology via some simple and low-cost techniques are highly critical for superior electrochemical performances of TiO_2_-based supercapacitors for commercial charge storage devices.

Hydrothermal process, which is a simple and cost-effective method, has been used extensively to fabricate various titania/titanate nanostructures^27,28^. Especially, the alkali-controlled hydrothermal treatment of titania is found to be highly effective to control the surface morphology of titania/titanate nanostructures^28^. But for electrochemical device-related applications, the interfacial contact between the nanostructured titania and metal electrode is crucial, which is, in general, modeled in terms of Schottky barrier, manifested by the Fermi level mismatch between the semiconducting titania and metallic electrode^29^. Lesser the interfacial defect is, higher is the direct electrical pathways of charge carriers between the titania-metal interface to improve the charge transport properties, and hence, an enhancement in the electrochemical performance. But, in majority of cases, the as-synthesized titania nanomaterials (step-1) are deposited by some conventional top-down approaches (step-2) onto the metal/metal-coated substrates to fabricate the electrochemical electrodes^30,31^. This, two-step process sometimes leads to the formation of interfacial defects to deteriorate the electrochemical (and similar interfacial) properties of the samples. To overcome this, titania nanotube arrays are fabricated directly onto the Ti foils via conventional anodization technique^8–10,12,21–23^, which are then used as the electrochemical electrodes for supercapacitors. But the anodization technique, which is a solution-based process, has some intrinsic disadvantages^32,33^, such as the (i) formation of a barrier oxide layer, (ii) creation of structural defects, (iii) incorporation of impurities, etc. at the oxide-metal interface, which hinder the charge transport properties between the TiO_2_-Ti interface, and hence, affect the electrochemical properties.

The above observations indicate the necessity of alternative synthesis routes to fabricate interfacial-defect-free titania electrodes for superior electrochemical properties. In that respect, recently, we have reported the *in-situ* surface modification of Ti substrate into titania/titanate nanostructures via an alkali-controlled hydrothermal technique, and showed the improved field emission properties^5^. By controlling the KOH concentration in the hydrothermal system, the surfaces of the Ti substrates have been systematically converted into 2D titania nanoplatelets and 1D nanorods/nanofibers structures. The *in-situ* surface modifications of the Ti substrate lead to the formation of well-adhered semiconducting titania nanostructures directly onto the metallic Ti, thus minimizing the interfacial defects, which played crucial role in the enhancement of the field-emission properties of the samples.

Therefore, using the similar synthesis protocol, as previously reported by us^5^, in the current manuscript, we have reported the formation of interfacial-defect-free TiO_2_ nanostructures via the *in-situ* surface reconstruction of a self-source Ti substrate through an alkali (KOH)-controlled hydrothermal process, and presented their superior electrochemical properties. A systematic study is conducted (for the first time, to best of our knowledge) to measure the electrochemical properties of the various titania nanostructures and observe the effect of the surface morphology on the supercapacitive performance of the nanostructured electrodes. Due to the unique structure of the electrode wherein the metallic surface is directly converted into oxide nanostructure via a bottom-up technique (instead of widely used top-down deposition approaches), direct electrical transport pathways for charge carriers increase the charge-transport rate^34^, which, in turn, improve the electrochemical performance of the electrode materials. Therefore, the specific advantage of this technique is that it can provide surface morphology-controlled self-assembled 1D/2D nanostructures of titania that are extremely well-anchored to the Ti-rich substrate, having excellent interfacial contacts between metal-metal oxide interface, resulting in superior charge transport properties for enhanced electrochemical (and similar device-related) performances.

Although, the similar synthesis protocol is reported by us previously^5^, but the importance of the current report is the use of the TiO_2_-Ti samples directly as the electrochemical electrodes, without any further modification. It is well-known that the electrochemical electrode preparation is a tedious yet important process for efficient electrochemical characterizations. Various methods (such as spin/spray coating, drop-casting, self-assembly, etc.) have been adopted to deposit the electro-active materials on the metallic or metal-coated substrates. Also, different carbon-based adhesives have been used as binders for high adhesivity of the active materials onto the metallic surface. Additionally, various physical and chemical treatments have been performed to the as-fabricated electrodes. All of these are attempted either to improve the stability of the electrode and/or enhance the electrical properties of the active material only^35–37^. But, surprisingly, none (to best of the authors’ knowledge) have attempted to specifically improve the interfacial properties of metal-metal oxide interface, which has profound effect on the charge transport properties of the electrochemical electrode. Therefore, the novelty of the current report is that, for the first time (best of our knowledge), *in-situ* surface modification is attempted to address the issues related to the interfacial properties of the TiO_2_-Ti electrodes for enhancement of the electrochemical performance. The novel idea of using the surface-modified Ti-TiO_2_ samples directly as the electrochemical electrode without using any additives, and/or without performing any surface modifications etc., is attempted to reduce the Ti-TiO_2_ interfacial resistance, and from electrochemical impedance spectroscopic studies, we have clearly observed much lesser charge flow resistance across this interface, which considerably improved the supercapacitive performance of the microelectrodes. And we have been able to obtain a maximum specific capacitance value around 7.4 mF cm^−2^, which is considerably higher than the literature value under similar electrochemical conditions (details are discussed later). Hence, this simple and cost-effective one-pot synthesis can be adopted efficiently for superior electrochemical supercapacitors and other diverse surface-related device applications.

## Hydrothermal Chemistry

Generally, the alkali-based hydrothermal reaction of Ti substrate follows two simultaneous reactions: dissolution of metallic Ti into Ti-hydroxides and growth of titania via the reaction of Ti-hydroxides with hydroxyl ions, and given by the following equations^38^:

1 $$Ti+4{H}_{2}O\to Ti\,{(OH)}_{3}^{+}+{(OH)}^{-}+2{H}_{2}\,(dissolution)$$

2 $$Ti\,{(OH)}_{3}^{+}+{(OH)}^{-}\to Ti{O}_{2}+2{H}_{2}O\,(growth)$$

Kinetically, the dissolution and growth will compete with each other, and depending on the hydrothermal conditions as well as the KOH concentrations, one would balance and/or dominate other.

**Figure 1:**
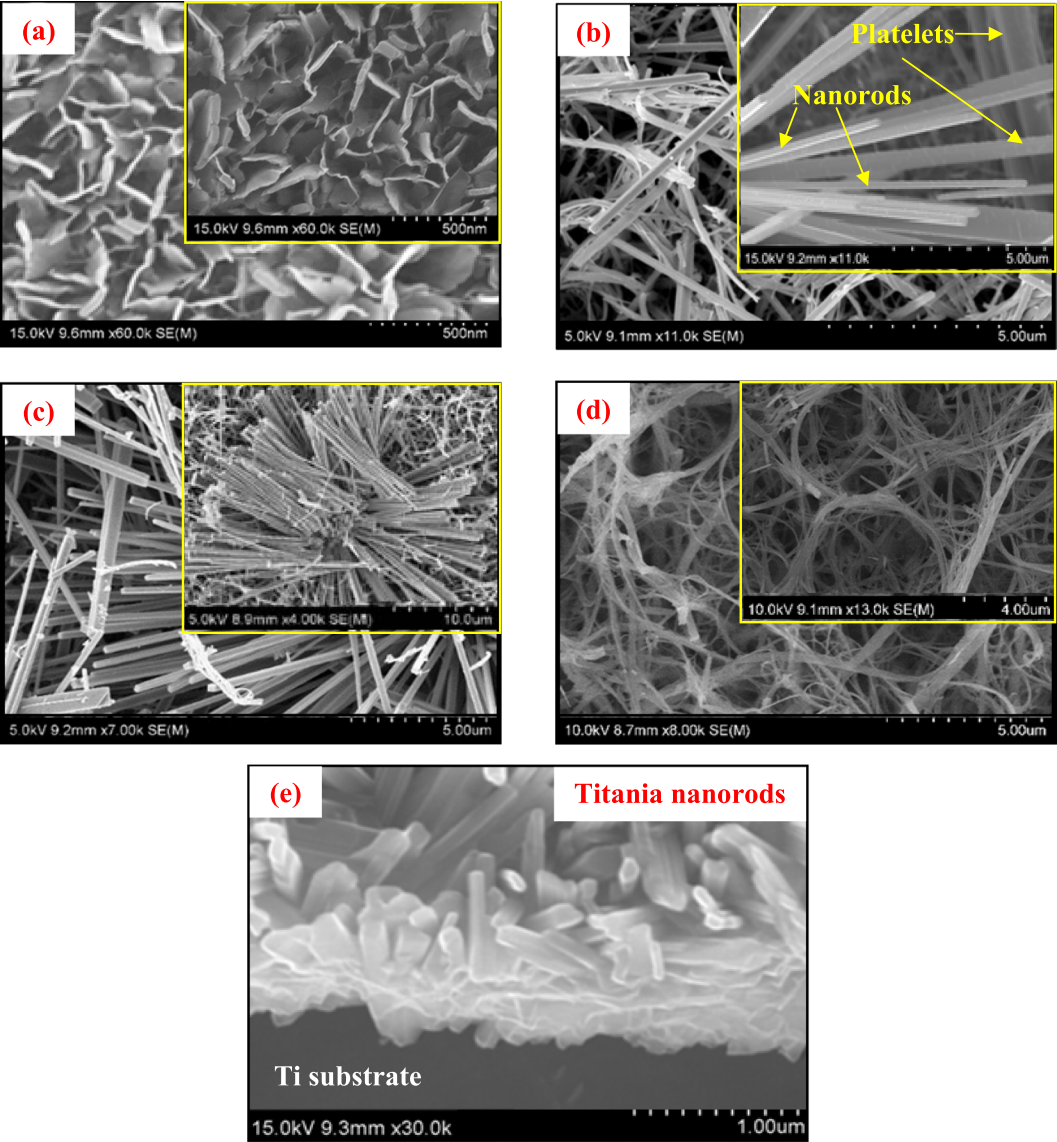
FESEM images of titania/titanate nanostructures directly synthesized on Ti substrate at 250 °C hydrothermal temperature at different KOH concentrations of (**a**) 0.25 M (2D platelets), (**b**) 0.5 M (2D platelets + 1D nanorods mixture), (**c**) 1.0 M (1D nanorods), (**d**) 5.0 M KOH (1D nanofibers), respectively. Insets represent the closer view of the nanostructures. (**e**) Cross-sectional view of the formation of titania nanorods directly onto the self-source Ti substrate. All samples are post-annealed at 500 °C in air.

As shown in Fig. S1 (also described in details in Fig. 1), according to the current experimental conditions, at low KOH concentration (0.25 M), oxide growth dominates the dissolution, resulting in the formation of some flower-petal-like 2D platelet structures. With the increase in the KOH concentrations to 0.5 M, dissolution increases and starts competing with the growth, resulting in the formation of a mixed morphology of 2D platelet and 1D nanorod structure. When the KOH concentration is further increased to 1.0 M, dissolution dominates the growth and a highly oriented 1D nanorod structure is obtained. Finally, when the KOH concentration is increased to as high as 5.0 M, dissolution becomes considerably higher than the growth and the 1D nanorod structure is further reduced to much thinner nanofibrous structure.

Additionally, the KOH-controlled hydrothermal modification of metallic Ti also involves the formation of some K-polytitanates. This is manifested by the dissolution of Ti into the aqueous KOH solution to form some polytitanate anions, and, depending on the hydrothermal conditions as well as the KOH concentration, these polytitanate anions are then precipitated in the form of K-polytitanates onto the titania surface. This K-polytitanate formation can be represented as^38,39^:

3 $$2mKOH+2nHOH\to 2m{K}^{+}+2(n+m)O{H}^{-}+2n{H}^{+}\,({\rm{hydrolysis}})$$

4 $$\begin{array}{c}nTi+n{H}^{+}+(n+m)O{H}^{-}\to {[T{i}_{n}{O}_{(n+m)}]}^{(m)-}+(\frac{2n+m}{2}){H}_{2}\\ (\mathrm{polytitanate}\,{\rm{anion}}\,\mathrm{formation})\end{array}$$

5 $$\begin{array}{c}2m{K}^{+}+{[T{i}_{n}{O}_{(n+m)}]}^{(m)-}+(n+m)O{H}^{-}\\ \quad \quad \,\,\,+\,n{H}^{+}\to {K}_{2m}{O}_{m}\bullet nTi{O}_{2}\bullet m{H}_{2}O+(\frac{2n-m}{2}){H}_{2}\\ \quad \quad \quad \quad \quad \quad (\mathrm{Hydrated\; K}-{\rm{polytitanate}}\,\mathrm{precipitation})\end{array}$$

Upon heating, the hydrated K-polytitanate (*K*_*2m*_ *O*_*m*_ • *nTiO*_2_ • *mH*_2_ *O*) is converted to potassium-polytitanate (*K*_2*m*_ *Ti*_*n*_ *O*_*2n+m*_). Apparently, both the titania and K-polytitanate formations occur simultaneously, and in the current case, with the increase in the KOH concentrations, K-polytitanate formation is found to increase, which is corroborated by the XRD measurements and explained in details later. Because of the low water solubility, K-polytitanates precipitate on the titania surfaces via heterogeneous deposition^5^.

## Results and Discussion

### SEM Analysis

Figure 1 represents the SEM micrographs of the surface morphology of titania/titanate nanomaterials fabricated at different KOH concentrations of 0.25 M, 0.5 M, 1.0 M and 5.0 M, respectively. Evidently, the KOH concentration within the hydrothermal environment is found to have profound effect on the surface structures of the Ti substrate. With increase in the KOH concentration, a dimensional reduction of the titania surface nanostructure is manifested according to the dissolution and growth reactions shown in Eqs 1 and 2, and demonstrated in Fig. 1. For example, at 0.25 M KOH, scaffolds of 2D platelet structures (Fig. 1a) are produced with a nanoplate size range around 250 to 450 nm. At 0.5 M KOH, 2D nanostructures are started to reduce into 1D nanorods, resulting in a mixture of 1D nanorod + 2D platelet nanostructure (Fig. 1b) with a rod thickness roughly ranging around 200 to 300 nm and plate size around 250 to 400 nm. At 1.0 M KOH, entire 2D platelets are completely converted into well-defined 1D titania nanorods (Fig. 1c) with wide thickness variations from less than 100 nm to more than 400 nm. Finally, at 5.0 M KOH, nanofibers of titania are formed (Fig. 1d) with thickness roughly ranging around 100 to 300 nm.

Also, the direct conversion of the metallic Ti substrate into titania nanostructures is shown in the cross-sectional SEM image in Fig. 1(e). This clearly verifies the importance of the current fabrication technique, where the self-source Ti substrate surface is directly reconstructed into morphology-controlled metal oxide nanostructures with reduced interfacial defects for superior electrochemical and other device-related applications.

### XRD Analysis

**Figure 2:**
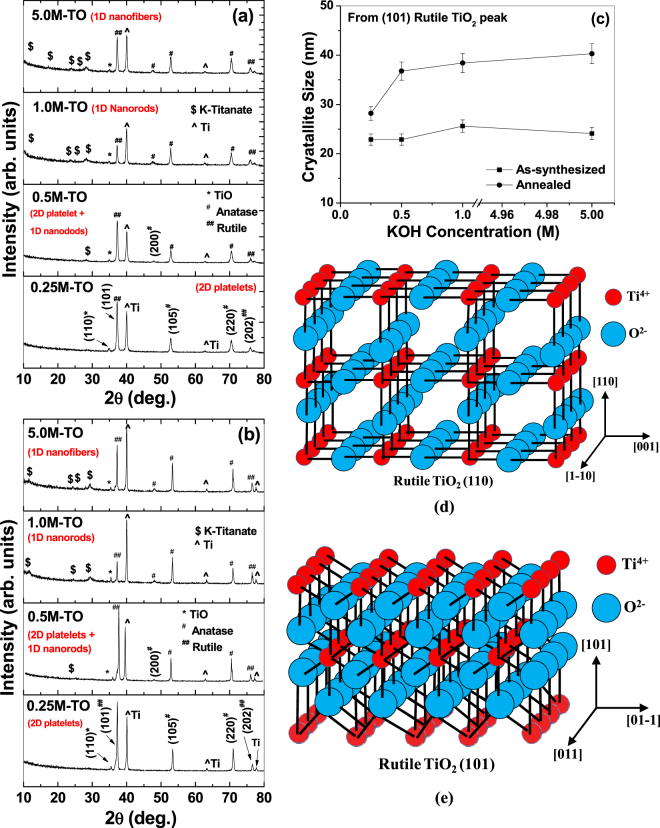
XRD pattern of hydrothermally prepared titania nanostructures (**a**) before air-annealing, and (**b**) after air-annealing at 500 °C. (**c**) Crystallite size as a function of KOH concentration for un-annealed (lower curve) and air-annealed (upper curve) samples. (**d**,**e**) Crystal structures of the (110) and (101) planes of Rutile TiO_2_ (not to scale). *Figure* (*d*,*e*) *is adapted with permission from Yong Li and Wenjie Shen, Chem. Soc. Rev. 43 (2014)* *1543. Copyright (2014)* *Royal Society of Chemistry*.

Structural analyses of the nanomaterials are shown in Fig. 2. The as-synthesized samples at different KOH concentrations, before annealing, and after-air-annealing (@500 °C), are shown in Fig. 2(a) and (b), respectively. In both cases, the XRD graphs depict a mixture of anatase (marked # in the figures) and rutile titania (marked ## in the figures) as well as a small fraction of TiO phase (marked * in the figures). All the Ti peaks (marked **^** in the figures) are originating from the metallic substrate. A comparison of the 2*θ*-values of all the available peaks with the 1999 Joint Committee on Powder Diffraction Standards - The International Centre for Diffraction Data (JCPDS-ICDD) File Cards has been presented in Table S1 (Supplementary Data). It must be mentioned in this connection that, according to the 1999-JCPDS-ICDD File card # 78-1510, the most dominant rutile peak is around 27.5° of 2*θ*-value (for rutile 110 reflection with *d*-value = 0.324 nm), but one of the next dominant (and widely reported) peaks is around 36.2° of 2*θ*-value (for rutile 101 reflection with *d*-value = 0.248 nm). In our XRD data, we have observed this second dominant peak of rutile (101) reflection around 36.8° of 2*θ*-value, which is matching well with the JCPDS file card # 78-1510 (as clearly presented in Table S1, Supplementary data). Although we have not observed the most dominant peak (rutile 110 around 27 ° of 2*θ*) in the XRD curve, but we have been able to observe the second dominant peak (rutile 101 around 37° of 2*θ*) as the highest rutile peak in the XRD pattern. Because of the controlled atmosphere in a hydrothermal system with very high pressure and temperature (as in the current case), sometimes the most dominant peak is suppressed in the XRD graphs whereas lesser dominant peaks are evolved more due to the available energy conditions within the hydrothermal system. For example, Gardecka *et al*.^40^ reported that, in the XRD pattern of their hydrothermally grown rutile titania, the most dominant (110) peak (at 27° of 2*θ*) has been suppressed considerably, whereas the rutile (101) peak (at 37 ° of 2*θ*) has become the highest peak. This result is similar to our data.

**Figure 3:**
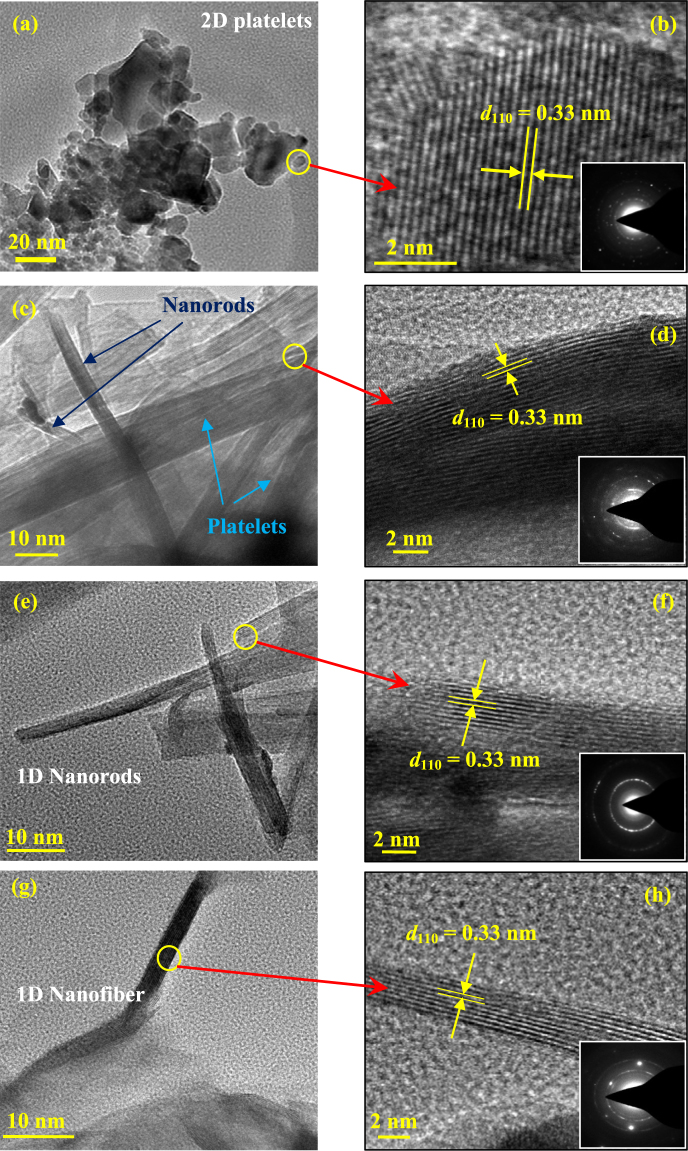
TEM (**a**,**c**,**e**,**g**) and HRTEM (**b**,**d**,**f**,**h**) images of titania nanostructures hydrothermally created on self-source Ti substrates at 0.25 M, 0.5 M, 1.0 M and 5.0 M KOH concentrations, respectively. Proper lattice spacings of (110) planes of rutile TiO_2_ is clearly visible. Insets of (**b**,**d**,**f**,**h**) represent corresponding SAED patterns. All samples are post-annealed at 500 °C in air.

A comparison between Fig. 2(a) and (b) (having identical *y*-axes scales) reveals that the peak intensities are increased considerably under annealing, which is in the expected line, since the crystallinity of the samples is increased under thermal treatment. Apart from the changes in the peak intensity and peak width, no other drastic changes in the crystalline phases are observed against the variations in KOH concentrations as well as annealing. This indicates that the current alkali-based hydrothermal process mainly controls the morphology of the sample surfaces, without changing the crystalline phases and chemical compositions of the nanomaterials. To further corroborate this fact that these changes are basically physical in nature, crystallite size calculations (by Scherrer formula, as explained in the Experimental section) are performed for (101) rutile peaks as a function of KOH concentrations as well as the annealing treatments, and presented in Fig. 2(c). For un-annealed samples, crystallite size is found to be near-constant (within 5%, as shown by the error bars in the lower curve) at different KOH concentrations. As obvious, with annealing, the crystallite size increases considerably (upper curve) due to the fact that the thermal energy favors the agglomeration of smaller crystallites into bigger ones. Also for the annealed samples, the crystallite size is found to increase considerably (from 28 nm to 37 nm) when the KOH concentration increases from 0.25 M to 0.5 M, and then remains near-constant (within 5%, as shown by the error bars in the upper curve) at higher concentrations. This is mainly due to the morphological transformation of the samples from 2D platelet structure to 1D nanorod structure. Thermodynamically, when the growth is restricted along a certain direction, the crystal growth is preferred along other direction(s) which is(are) crystallographically favored. In the current case, when the 2D platelets are dimensionally reduced to 1D nanorods by increasing the KOH concentrations, TEM images in Fig. 3(d,f,h) and Fig. S2 depict that the nanorods/nanofibers grow in a direction parallel to the Rutile (110) plane (i.e. perpendicular to the [110] direction) at the edges and along (101) plane at the interior (details are discussed later). The crystal structure of (110) and (101) planes of Rutile TiO_2_ is schematically shown in Fig. 2(d) and (e), respectively. Generally, these are the two dominant peaks of Rutile structure, and depending on the growth conditions and favorable energy conditions, either (or both) grow preferentially. In the current case, as the crystal growth is favored along the directions parallel to the (101)/(110) planes (at the interior/edge, respectively), the domains of the crystallites tend to merge along these directions to produce bigger crystallites against 2D structures^41^.

**Figure 4:**
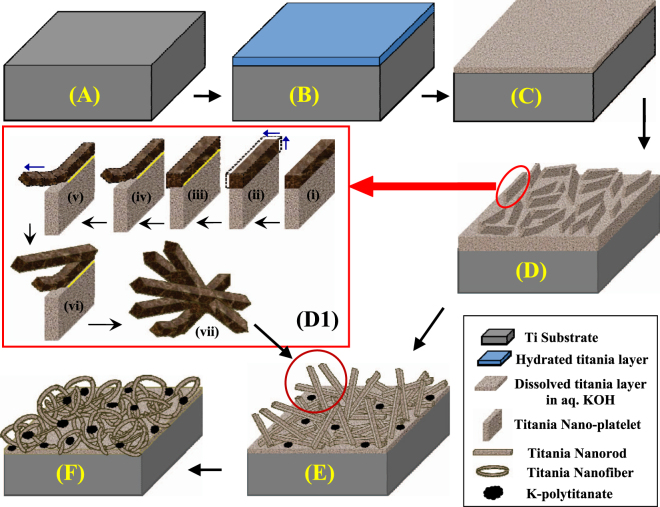
Schematic diagram of the hydrothermal surface modification of self-source Ti substrates to form various 2D and 1D surface nanostructures. The diagram is not to scale. *The* figure *D1 is adapted with permission from V C Anitha et al. “Morphology-dependent low macroscopic field emission properties of titania*/*titanate nanorods synthesized by alkali-controlled hydrothermal treatment of a metallic Ti surface”, Nanotechnology 26 (2015)* *355705. ©* *(2015) IOP Publishing. All rights reserved* [doi:10.1088/0957-4484/26/35/355705].

Additionally, traces of K-polytitanate peaks are also observed within the XRD patterns (marked $ in the figures), which is due to the precipitation of polytitanates according to Eqs 3–5. With increase in the KOH concentrations, the number of K-polytitanate peaks is also increased, indicating that the polytitanate formation is an increasing function of the KOH concentration. This is because, according to Eqs 3–5, K-polytitanate formation occurs during the dissolution of hydrated titania layer into aqueous KOH (*cf*. Fig. 4B–D). Kinetically, higher concentration of KOH increases the formation rate of dissolved titania layer into KOH, leading to the formation of more K-polytitanates over a certain period of time^32^. Also, when the as-synthesized titania/titanate nanostructures are air-annealed, some of the titanate peaks are either disappeared or reduced, which is due to the partial conversion of the titanates into titania, as reported by others too^42,43^.

### Compositional Analysis

EDX analyses of the four samples are presented in Fig. S3. Presence of elemental Ti and O are clearly evidenced, alongwith some amount of K. The elemental percentages are tabulated at the corresponding insets. The presence of elemental K is due to the precipitation of K-polytitanate, as explained in Eqs 3–5. Although, at 0.25 M KOH concentration, K-polytitanate formation is negligible to be detected, but at higher KOH concentrations, K-polytitanate formation increases, indicating that the K-polytitanate formation is highly dependent on the KOH concentration. Also, all the samples are found to be considerably nonstoichiometric in terms of excess oxygen, which is manifested due to the presence of polytitanates as well as other phases (such as TiO, rutile and titania) within the nanostructures, as evidenced in the XRD data.

### TEM Analysis

The microstructures of the titania nanomaterials are shown in Fig. 3. Figure 3(a,c,e,g) represent the TEM micrographs of 2D platelets/1D+2D mixture/1D nanorods/1D nanofibers, respectively. Corresponding HRTEM images and selected area electron diffraction (SAED) patterns are shown in Fig. 3(b,d,f,h) and insets, respectively. Well-aligned (110) lattice planes of rutile titania (which is the most dominant reflection, according to the 1999 JCPDS-ICDD File Card # 78-1510, *cf*. Table S1), with proper *d*-spacings (~0.33 ± 0.01 nm) are clearly observed, indicating high crystallinity of the samples. Interestingly, this lattice spacing is observed mainly at the edges of the nanoplatelets and nanorods/nanofibers. But, at the interior of the nanoplatelets/nanorods, the lattice spacing resembles more with the Rutile (101) reflection (as shown in Fig. S2, Supplementary Data and compared with 1999 JCPDS-ICDD File Card # 78-1510 in Table S1), which is also substantiated by the XRD data. Since TEM is a localized characterization process, we have been able to observe the growth of rutile (110) planes at the edges of the samples, although it is missing in the XRD graphs. Since the fraction of this lattice planes at the edges is considerably smaller than that of the rutile (101) planes at the interior of the nanomaterials, the powder diffraction intensity becomes too low for the (110) reflection, and therefore masked by the background noise, and hence cannot be detected in the XRD graphs. This type of growth of different crystal planes at the edges and at the interior of hydrothermally grown metal oxide nanocrystals have already been reported by others^44^. For titania nanocrystals also, similar types of growth of different crystal planes at different facets of the nanocrystals have been reported too^45^. The reason for this is mainly the growth condition that leads to the favorable energy condition for the preferential growth of one plane over the other. As shown in Fig. 2(d,e), the two dominant crystal planes of Rutile structure are generally favored, and in the current case, under the applied hydrothermal conditions, rutile (110) planes are energetically favored at the edges, whereas at the interior of the nanocrystals, due to space restriction, rutile (101) planes grow preferentially.

### Mechanism

Figure 4 represents the schematic description of the formation mechanism of titania/titanate nanostructures on the self-source Ti substrate under KOH-controlled hydrothermal process^5^. Initially, the surface of the metallic Ti substrate (Fig. 4A) reacts with the aqueous KOH to form a thin layer of hydrated titania (Fig. 4B), which is then transformed into a dissolved titania layer into aqueous KOH (Fig. 4C). Thereafter, depending on the hydrothermal conditions and KOH concentrations, dissolution and growth reactions (Eqs 1 and 2) are manifested, resulting in the formation of 2D platelets (Figs 1d and 4D)/nanorods (Fig. 4E)/nanofibers (Fig. 4F), respectively, as an increasing function of KOH concentrations.

Interestingly, the 2D platelet structures are converted into 1D nanorod structures via some intermediate conversion steps, which are schematically depicted in Fig. 4D1. After the 2D platelet formation (at 0.25 M KOH), the edges of the platelets, which are exposed to the reaction environment, start to accumulate some excess oxides via a dominant growth reaction, thus converting the edges into more thickly than the interior of the platelets (4-D1-i). This is clearly verified by the SEM images shown in Fig. 1(a), where the edges of the platelets appear to be thicker than the interior. With propagation of the reaction time, the edges of the platelets grow farther along the width (as shown by the blue arrows in 4-D1-ii), and due to the mass difference between the less massive inner portion of the platelets and more massive edges, a line of strain evolves along the interface of these two portions (shown by the yellow line in 4-D1-iii). With further increase in the KOH concentration (to 0.5 M), the etching rate increases. This makes the line of strain energetically unstable, and hence, the massive rod-like edges start to tear off from the platelets, thus initiating the formation of 1D nanorods (4-D1-iv). This is again substantiated by the SEM image shown in Fig. 1(b), where some tearing-off of the rod-like structures is observed. With further increase in the KOH concentration to 1.0 M, the etching rate increases further, which leads to the growth of 1D nanostructures into longer and thinner nanorods, as shown by blue arrow in 4-D1-v, and also verified by the SEM image in Fig. 1(c). After tearing off the nanorods from the platelets, further growth of the mass occurs at the edges of the remaining platelets followed by subsequent strain generation and nanorod formation (4-D1-vi) in a similar manner, as discussed above. This process continues until all the platelets are transformed into bundles of nanorods, as shown schematically in 4-D1-vii and corroborated by the SEM image in the inset of Fig. 1(c). Thus the metallic Ti substrate surface is converted into 1D titania nanorods, as shown schematically in Fig. 4E, and also substantiated by the SEM image in Fig. 1(e). Finally, at very high KOH concentrations (5.0 M), the 1D nanorods are further dimensionally reduced to 1D nanofibers, as shown in Fig. 4F and verified by the SEM image in Fig. 1(d). Additionally, according to Eqs 3–5, some K-polytitanates are also formed and precipitated on the titania nanostructures, as schematically shown in Fig. 4E,F, and corroborated by the XRD data, discussed above.

### XPS Analyses

#### Survey Spectra

**Figure 5:**
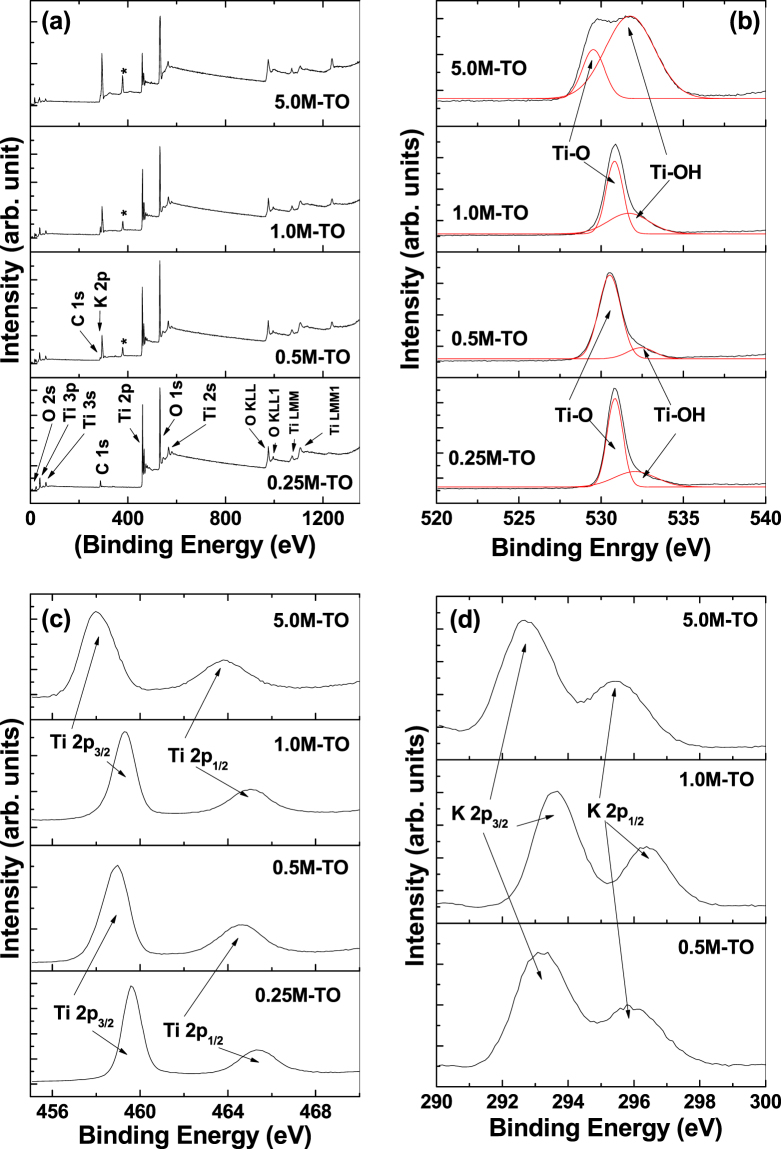
XPS survey (**a**), O 1 s (**b**), Ti 2p (**c**), and K 2p (**d**) spectra of hydrothermally-synthesized titania nanostructures at different KOH concentrations of 0.25 M (2D platelets), 0.5 M (2D platelets + 1D nanorods mixture), 1.0 M (1D nanorods) and 5.0 M (1D nanofibers), respectively. The peak marked in * at (**a**) is assumed to be due to Ag 3*d* state, the origin of which is explained in the text in details.

The chemical composition and binding energy of the titania/titanate nanostructures are studied through XPS analyses (Fig. 5). Figure 5(a) represents the XPS survey spectra of all the samples, whereas Fig. 5(b,c,d) represent the corresponding high-resolution spectra of O 1*s*, Ti 2*p* and K 2*p* spectra of titania/K-titanate nanomaterials, respectively, fabricated at different KOH concentrations. The presence of elemental O, Ti and K are evidenced in the survey spectra. Tracer amount of the presence of C is due to the atmospheric contaminations during sample preparation and handling. Absence of the K 2*p* peak within the sample prepared at 0.25 M KOH is consistent with the EDX and XRD spectra (Figs S3 and 2). Apart from the elemental peaks, some Auger characteristic peaks (O KLL, Ti LMM etc.) are also observed, all of which are consistent with the previous reports^46,47^. Additionally, one unknown peak is observed around 374–378 eV (marked *), which closely resembles with Ag 3*d* peak^48^. It is surprising to see this peak since no Ag-source is used during hydrothermal reaction. Therefore, we believe that the origin of this peak is due to some Ag-contaminants within the Ti disc, as commercially available titanium often contains various contaminants including Ag^49^. This conclusion seems reasonable as the intensity of this peak is found to increase with increase in the KOH concentrations. This implies that with higher KOH concentrations during hydrothermal process, more etching of the Ti surface is manifested, leading to the more exposure of the Ag-contaminants. Probably the amounts of these contaminants are too low to be detected in EDX and XRD measurements. Also these Ag-contaminates at the titania nanostructured surfaces appear to play some role during the electrochemical activities of the titania electrodes, which is explained later.

#### O 1s Spectra

The high resolution O 1 *s* spectra for all the samples (presented in Fig. 5b) are fitted with two components. The peak at binding energy (BE) 530 ± 0.5 eV is attributed to the oxides (O^2−^) of the Ti-O bonds, whereas the other peak at BE 532 ± 0.5 eV is originating from the surface hydroxyl groups in the form of Ti-OH bonds^50^. For the sample prepared at 5.0 M KOH concentration, this Ti-OH peak is found to increase considerably, whereas the corresponding Ti-O peak is shifted slightly to lower BE value, against the other samples. This is mainly because of the use of very high concentration of KOH solution (5.0 M), which leads to the modification of the chemical environment of the nanomaterial, leading to the formation of high percentage of surface hydroxyls.

#### Ti 2p Spectra

The high-resolution Ti 2*p* spectra (Fig. 5c) of all the samples show two peaks at 458 ± 1.0 eV and 464 ± 1.0 eV, which are attributed to Ti^+4^ 2*p*_3/2_ and Ti^+4^ 2*p*_1/2_ states of photoelectrons, respectively^8,50^. Similar to the O 1 *s* spectra, for the sample prepared at 5.0 M KOH concentration, both the Ti 2*p* peaks are found to shift towards lower BE values, which is again attributed to the change in the chemical environment of the samples due to the use of high concentration of KOH solution (5.0 M). In general, the exact binding energy of an electron not only depends on the level from which the photoemission is occurring, but also on the local chemical environment (i.e. local bonding environment), which is affected by the formal oxidation state of the species, presence of neighboring atoms etc, that leads to variations in the coulombic interaction between the photoemitted electron and the ion core, and hence, a chemical shift occurs^51^. In the current case, the EDX data (Fig. S3) revealed the presence of excess elemental K (in the form of K-polytitanate) for the sample prepared at 5.0 M KOH concentration, which probably lead to the change in the local bonding environment, and hence a change in the chemical environment of the sample to manifest the chemical shift.

#### K 2p Spectra

High-resolution K 2*p* spectra (shown in Fig. 5d) for all the samples (except the one prepared at 0.25 M KOH) are fitted with two components at BEs 293 ± 0.5 eV and 296 ± 0.5 eV, and attributed to the K^+^ 2*p*_3/2_ and K^+^ 2*p*_1/2_ states of photoelectrons, respectively, which are originated from the spin-orbit doublet of potassium ions in oxides^52,53^. Again the slight low-BE shift of the sample prepared at 5.0 M KOH against the other samples is manifested by the change in the chemical environment for higher concentration of KOH solution.

From the XPS spectra it becomes apparent that the samples do not contain any Ti^+3^ (Ti^+3^ 2*p*), metallic K (K° 2*p*), surface chemisorbed/physisorbed water molecules (H-OH) and/or oxygen vacancy related states^8,11,53^. This is consistent with the electrochemical data (shown below), where very little pseudocapacitive behavior is observed within the samples, which generally occurs due to the presence of some of the above states.

### Electrochemical Properties

#### CV Analyses

**Figure 6:**
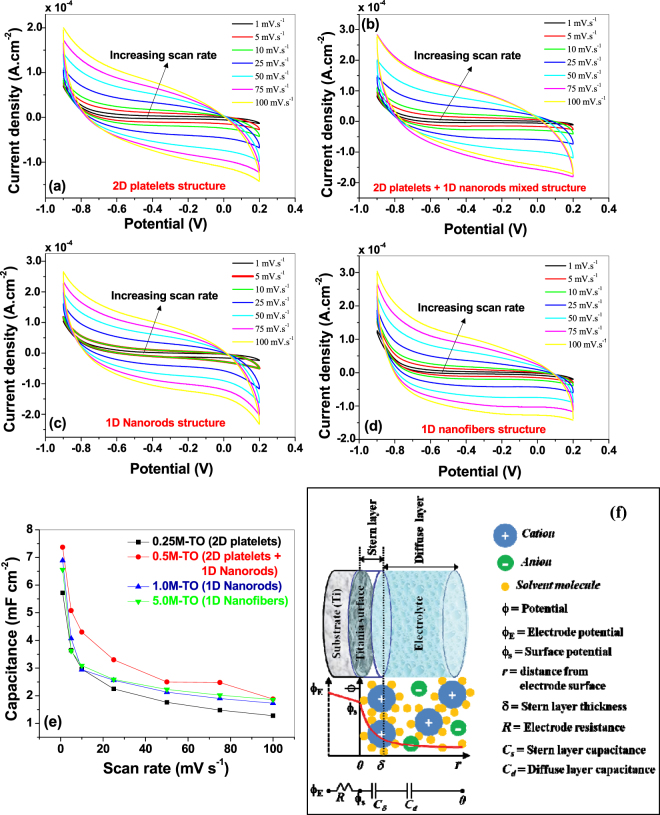
Cyclic voltammograms of hydrothermally synthesized titania nanostructures at different scan rates for (**a**) 0.25 M, (**b**) 0.5 M, (**c)** 1.0 M and (**d**) 5.0 M KOH solutions. (**e**) Comparison of the capacitance values of all the titania samples as a function of scan rates, calculated from the CV data. (**f**) Schematic diagram of the electric double layer formation between the titania – electrolyte interface, and corresponding potential variation across the electrode-electrolyte system, and the related equivalent circuit representation. *Figure* (*f*) *is adapted with permission from Hainan Wang, Laurent Pilon, “Physical Interpretation of Cyclic Voltammetry for Measuring Electric Double Layer Capacitances”, Electrochimica Acta 64 (2012) 130–139. © (2012) Elsevier Ltd*.

The first method used to determine the electrochemical performances of the titania/titanate – Ti electrodes for supercapacitor applications is the cyclic voltammetry (CV). The CV response curves at different scan rates (@ 1, 5, 10, 25, 50, 75, 100 mV s^−1^) for the samples prepared at different KOH concentrations are presented in Fig. 6(a–d). The curves show quasi-rectangular shapes, which is typical of the electrochemical double layer capacitance (non-faradaic)^21^. In all the samples, no distortion in the CV curves is observed, indicating strongly adhered titania nanostructures on the Ti substrate with highly efficient interlayer charge transfer capability, as well as no oxide layer breakdown at the specified scan rates. The specific capacitances (*C*_*s*_) of the samples are calculated from the CV curves according to following equation^37^ and presented in Fig. 6(e).

6 $${C}_{s}=\frac{1}{A\nu ({V}_{2}-{V}_{1})}{\int }_{{V}_{1}}^{{V}_{2}}I(V)dV$$

Here *ν* (V s^−1^) is the scan rate, (*V*_*2*_ − *V*_1_) is the potential window, *A* (cm^2^) is the area of the working electrode. The calculated *C*_*s*_-values at 1.0 mV s^−1^ scan rate for all the samples are compared in Table 1. The sample prepared at 0.5 M KOH solution showed the best capacitive performance against the other samples with a maximum *C*_*s*_ value around 7.4 mF cm^−2^. This value is considerably higher than the previously reported values of titania nanostructures calculated from the CV curves (0.01–2.6 mF cm^−2^) under similar scan rates^14,15,19,21,23^. For example, Salari and co-authors^23^ reported the *C*_*s*_-value for titania nanotubes (grown on Ti substrate) obtained from CV data (using 1 M KCl as active electrolyte, Ag/AgCl as reference electrode, and at a scan rate of 1.0 mV s^−1^, which is similar to the current report) around 0.9 mF cm^−2^. This group observed an increment in the *C*_*s*_-value upto 2.6 mF cm^−2^ (using 1 M NaOH aqueous solution as active electrolyte, Ag/AgCl as reference electrode, and at a scan rate of 1.0 mV s^−1^) of the titania nanotube arrays via a controlled phase transformation, which is still lesser than the current report. We have also reported the enhancement in the *C*_*s*_-value of titania nanotube arrays (by inducing nonstoichiometry) upto 5.5 mF cm^−2^ (under similar CV conditions as current report)^8^, which is lesser than that reported here. Also, Fig. 6(e) reveals a decrease in the capacitance values with the increase in the scan rates, which is due to the diffusion limitations of the charge carriers in the nanostructured surfaces at higher scan rates^21,24^.

**Table 1:** Specific capacitance (*C*_*s*_) values of all the samples calculated from CV and GCD data.

Samples	*C*_*s*_ (mF cm^−2^)				
	From CV data			From GCD data	
	*@1.0 mV s* ^−*1*^ *scan rate*	*@50.0 mV s* ^−*1*^ *scan rate*	*@100 mV s* ^−*1*^ *scan rate*	*@100 μA cm* ^−*2*^ *current density*	*@500 μA cm* ^−*2*^ *current density*
**0.25M-TO** (*2D platelets*)	5.72	1.76	1.28	1.83	1.09
**0.5M-TO** (*2D platelets* + 1*D nanorods*)	**7.37**	**2.50**	**1.88**	**2.64**	**1.48**
**1.0M-TO** (*1D nanorods*)	6.89	2.13	1.73	2.15	0.86
**5.0M-TO** (*1D nanofibers*)	6.56	2.23	1.86	1.73	0.94

The superior electrochemical properties of the current samples against the previously reported values is mainly because of the novel fabrication technique used, where the self-source Ti substrate is directly converted into oxide nanostructures, resulting in near-defect-free Ti-TiO_2_ interfaces for superior charge transfer, as the conductivity and charge-transfer rate of the active material profoundly affect the super-capacitive properties of nanomaterials^12,54^. On the other hand, the higher specific capacitance of the sample prepared at 0.5 M KOH solution (2D platelet + 1D nanorod mixture sample) against the other samples is mainly due to two reasons: (1) higher active surface sites for better utilization of the active material, (2) presence of lesser amount of insulating/semiconducting K-polytitanate precipitates, that hinder very little to the charge transfer from electrolyte to the electrode. More details about the above two reasons are discussed below.

As far as the relation between the active surface sites of the electroactive material and the electrochemical performance is concerned, it is well-known that an electrochemical double layer is formed at the electrode-electrolyte interface of the non-faradic capacitors, which is schematically shown in Fig. 6(f). A thin layer of solvated ions is formed near the surface of the active material (called Stern/compact layer), through which charges are transferred from the electrolyte to the working electrode (via reversible ion adsorption through electrostatic interactions). Beyond this layer, a diffuse layer is created, where mobile ions are attracted towards the Stern layer under the combined influence of electrostatic forces and diffusion. Due to this, the potential across the electrode-electrolyte interface is drastically varied, as shown schematically in the figure, and also represented by an equivalent circuit consists of a series combination of electrode resistance (*R*), stern layer (*C*_*s*_) and diffuse layer (*C*_*d*_) capacitances, respectively^55^. Since the compact layers of ions, residing on the surface of the active materials of the electrode, contribute considerably to the capacitance, therefore, the effective surface area of the electrode becomes one of the key factors for the supercapacitive performance of the working electrode^56^. Generally, for nanomaterials, because of the low-dimensional structures, surface-to-volume ratio is considerably increased against their bulk counterparts. Also a dimensional reduction from 2D structure to 1D/0D structure(s) increases the surface-to-volume ratio. Additionally, nanostructuring of the surface also increases the surface roughness, all of which enhances the active surface sites of the electrodes for superior electrochemical performances^53^. In the current case, the AFM surface roughness measurements of 2D and 1D nanostructured samples are presented in Table 2, which reveals that the 2D platelet structure (fabricated at 0.25 M KOH) has highest surface roughness, whereas the 1D nanorod sample (fabricated at 1.0 M KOH) has least surface roughness values. For 2D platelet + 1D nanorod mixture sample (fabricated at 0.5 M KOH), the data are showing some ambiguous values. For example, when the AFM scan area involves more 2D structures than that of 1D nanorods, the *rms* roughness (*R*_*q*_) depicts a value close to 160 nm, whereas if the scan area involves more 1D nanorods, this value becomes close to 85 nm. Similar trend is followed for other roughness parameters (*R*_*a*_, *R*_*max*_) too. Due to this large difference of values that varies considerably from one scan area to another, we didn’t get any reasonable representative value of surface roughness for this sample, and hence, not presented in Table 2. In any case, considering the fact that higher surface roughness provides more active surface sites, the 2D platelet sample is expected to have highest specific surface area, and hence highest specific capacitance. But, as stated above, a dimensional reduction generally increases the surface-to-volume ratio, and therefore, 1D nanorod/nanofiber structures should also have higher specific surface area. A qualitative analysis is performed from the AFM data and SEM/TEM micrographs to estimate an approximate surface-to-volume ratio of the nanostructured materials, which is presented in Table 2. As predicted, nanorod/nanofiber structures showed higher surface-to-volume ratio than that of 2D platelet structures. Therefore, it becomes apparent that, due to the combinatorial effect of higher roughness in 2D platelets and higher surface-to-volume ratio in 1D nanorods, the 2D platelet + 1D nanorod mixture sample should have highest active surface sites. Although BET surface area measurements cannot be performed in our samples due to instrument limitations^57^, but previous reports corroborates this phenomenon^53^, where a nanorod + nanoparticle mixed powdered sample showed higher specific surface area than that of single phase (nanorods or nanoparticles) samples, indicating that in the current case, according to the qualitative analyses from surface roughness and surface-to-volume ratio data (discussed above), the 2D platelet + 1D nanorod mixture samples (fabricated at 0.5 M KOH) manifest higher active surface sites due to the combined effect of higher surface roughness at the 2D platelets and greater surface-to-volume ratio at the 1D nanorods. And hence, this sample showed higher specific capacitances against other samples (Fig. 6e).

**Table 2:** Various surface-related parameters for nanostructured titania samples prepared at different KOH concentrations.

Samples	*RMS* roughness (*R*_*q*_) (nm)	Average roughness (*R*_*a*_) (nm)	Maximum roughness (*R*_*max*_) (μm)	Average Surface-to-volume ratio (nm^−1^)
**0.25M-TO** (*2D platelets*)	162.93	119.74	1.489	0.53
**1.0M-TO** (*1D nanorods*)	85.31	65.56	0.619	1.00
**5.0M-TO** (*1D nanofibers*)	133.29	92.20	1.268	1.33

According to the above argument, 1D nanofiber structures (fabricated at 5.0 M KOH) should also show very high specific capacitance value against other samples, because of the considerably high surface roughness and greater surface-to-volume ratio at low-dimensional structures (Table 2). But XRD data (Fig. 2) revealed that this sample (as well as 1D nanorods samples, fabricated at 1.0 M KOH) contains considerable precipitates of K-polytitanates, which are low-conducting (insulating/semiconducting) in nature^39,43^, and hence hinders the charge transfer process to reduce the electrochemical performance against the 2D platelet + 1D nanorod mixed sample. This argument is validated by the conductivity measurements of the samples via a two-probe method under ambient conditions (conducting silver paste is used for ohmic contact, linearity of which is verified over a wide voltage range). The room-temperature conductivity (σ_RT_) values are presented in Table 3, which shows that 1D nanorods/nanofibers samples have lower conductivities than the 2D platelet samples. On the other hand, the 2D platelet + 1D nanorod mixed structure (fabricated at 0.5 M KOH), although contains a very small peak of K-polytitanate (Fig. 2), which slightly reduced the conductivity value (Table 3), yet compensated by the greater active surface sites (as discussed above) to show maximum specific capacitances against other samples.

**Table 3:** Room temperature conductivity (σ_RT_) and EIS fitted data for all the samples.

Samples	σ_RT_ (x 10^−3^ S m^−1^)	*R*_Ω_ (ohm)	*R*_ct_ (ohm)	CPE_DL_ (μF)	*ψ*_*w*_ (ohm s^−1/2^)
**0.25M-TO** (*2D platelets*)	2.48	1.32	1.94	5.5	615.6
**0.5M-TO** (*2D platelets* + *1D nanorods*)	2.43	5.68	0.82	12.0	1047.0
**1.0M-TO** (*1D nanorods*)	2.30	6.66	1.41	4.0	617.3
**5.0M-TO** (*1D nanofibers*)	1.73	5.02	1.65	2.2	493.2

#### GCD Analyses

**Figure 7:**
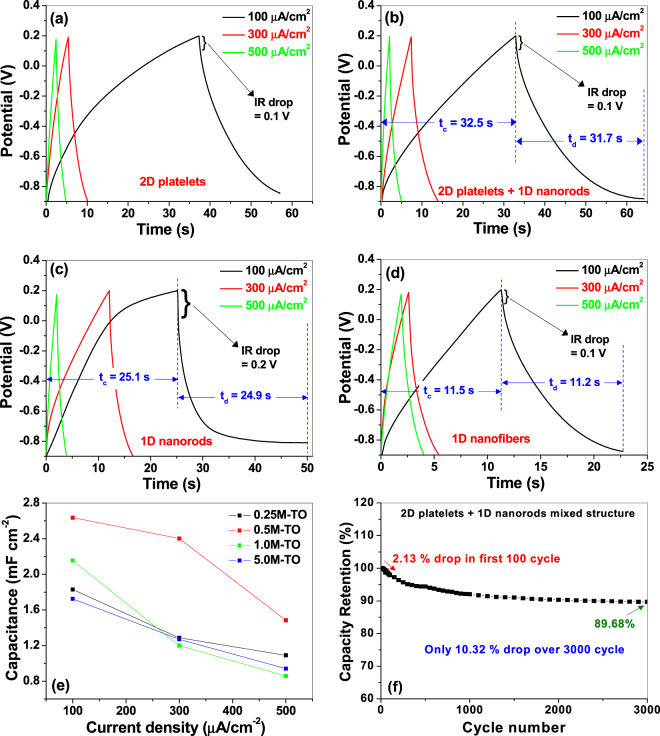
Galvanostatic charge-discharge curves at different current densities for titania nanostructures hydrothermally synthesized at (**a**) 0.25 M, (**b**) 0.5 M, (**c**) 1.0 M and (**d**) 5.0 M KOH solutions. (**e**) Specific capacitance as a function of current densities obtained from charge-discharge data for all the samples. (**f**) Cyclic stability plot for 2D platelets + 1D nanorods mixed sample.

The galvanostatic charge-discharge (GCD) curves for all the samples are presented in Fig. 7(a–d) over the same potential window as used in CV measurements, and at three different current densities (100, 300 and 500 μA cm^−2^). The negligible IR drops (0.1 V to 0.2 V, as shown in Fig. 7) in all the samples indicate potential applicability in high power devices^11,53^. Also the GCD curve profiles of all the electrodes depict slight deviations from the linear behavior, indicating some distinct energy storage mechanism and Faradaic pseudocapacitive reaction^52^. Although the CV curves (Fig. 6) reveal pure electric double layer capacitive activity, but we think some surface redox reactions have been manifested due to some surface states, which could not be detected during CV test. Similar phenomenon is also reported by others where CV test of TiO_2_ nanotube arrays depicted ideal double layer capacitive behavior, yet the GCD curves deviated from the linear characteristics^21,23^. This non-linearity in the GCD curve is predicted to be due to some surface impurity states in our sample. For example, XPS survey spectra (Fig. 5a) showed a probable Ag 3*d* peak, which is assigned to some surface Ag-contaminants within the nanostructured samples, and probably, these Ag-contaminants are involved in the surface redox reactions to show the pseudocapacitive behavior. This argument seems reasonable as the redox-active electrochemical behavior of Ag nanoparticles have already been reported by others^58^. Also, the charging and discharging times (*t*_*c*_/*t*_*d*_) in each electrode are found to be near-identical (as shown in Fig. 7), indicating high reversibility, with columbic efficiency (*η*% = *t*_*d*_/*t*_*c*_ × 100%^36^) of charge-discharge cycling very close to unity (*t*_*d*_/*t*_*c*_ ~0.97–0.99)^52^.

The specific capacitances (*C*_*s*_) of the samples are calculated from the GCD curves according to following equation^37^, and presented in Fig. 7(e).

7 $${C}_{s}=\frac{I{\rm{\Delta }}t}{{\rm{\Delta }}VA}$$

Here *I* (A) is the discharge current, Δ*t* (s) is the discharge time, Δ*V* (V) is the change in the potential during discharge and *A* (cm^2^) is the area of the working electrode. Corresponding *C*_*s*_ -values for all the samples, at a current density of 100 μA cm^−2^, are compared in Table 1. Similar to CV results, in this case also, the sample prepared at 0.5 M KOH solution (2D platelet + 1D nanorod mixture sample) showed the best capacitive performance against the other samples, which implies that the electrodes’ performance are consistent and highly reproducible in both cases. The maximum *C*_*s*_ value obtained for this sample is around 2.64 mF cm^−2^, which is considerably higher than the previously reported values of titania nanostructures calculated from the GCD curves (0.02–1.0 mF cm^−2^)^20,21,23^, under similar electrochemical conditions.

For practical applications of supercapacitors, long cycling life with high capacity retention is an important attribute for superior electrochemical performance. Figure 7(f) shows the capacity retention graphs for the 2D platelets + 1D nanorods mixed sample over 3000 cycles at a current density of 0.3 mA cm^−2^. As shown in the graph, only ~2.13% reduction in the specific capacitance value is observed for the first 100 cycles, and an overall meager decrement of 10.0% for the entire 3000 cycles, which clearly indicates that the as-synthesized titania nanomaterials can become very promising electrode material for high-rate charge/discharge operations in supercapacitors.

#### EIS Analyses

**Figure 8:**
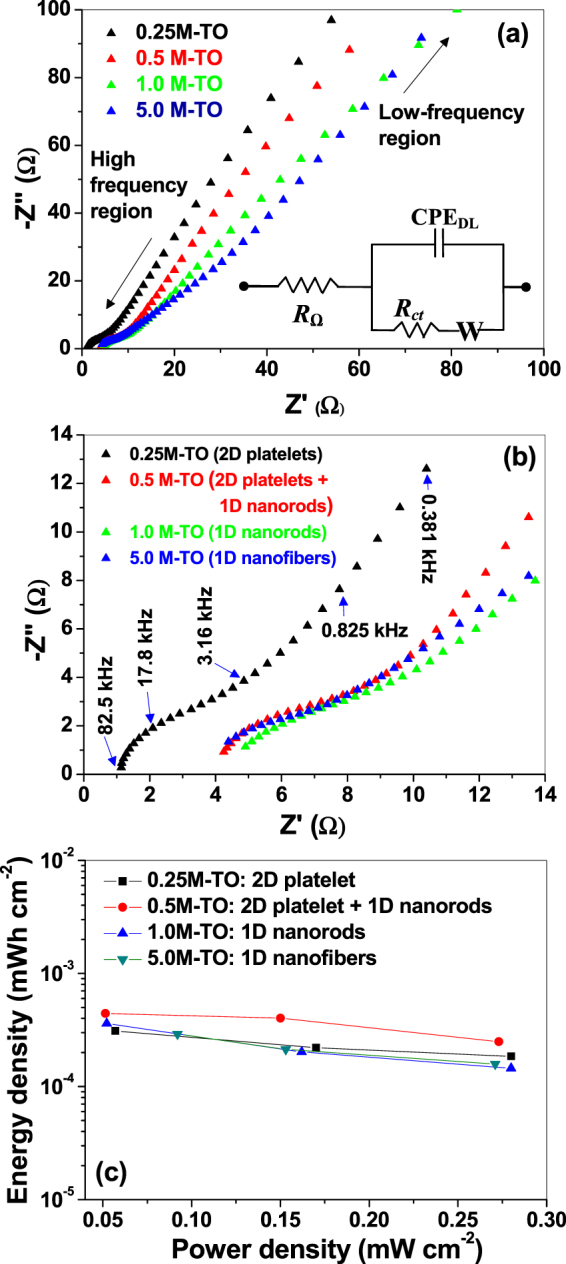
Nyquist plots of titania samples hydrothermally prepared at different KOH concentrations at (**a**) low-to-high frequency regions, (**b**) at high frequency region. Some of the frequencies are indicated in the graph. Inset of (**a**) represents the corresponding equivalent circuit. (**c**) Ragone plots of power density versus energy density calculated from the galvanostatic charge-discharge for all the samples.

To understand the kinetic properties of the nanostructured titania electrodes, electrochemical impedance spectroscopic (EIS) measurements are performed. Figure 8(a) represents the Nyquist plots of all the samples from low-to-high frequency regions, whereas the same are plotted only at the mid-to-high frequency regions in Fig. 8(b). All the plots consist of (*i*) a slightly depressed semicircle at the high-to-mid frequency regions and (*ii*) a linear part at the low frequency region. The semicircular part represents the Warburg curve^59^, which is a parallel combination of charge transfer resistance (*R*_*ct*_) and a double-layer capacitance (*CPE*_*DL*_). *R*_*ct*_ characterizes the redox reaction at the electrode-electrolyte interface and equal to the diameter of the semicircle, and *CPE*_*DL*_ represents the constant phase element (*CPE*) and occurs at the solid–liquid interface due to the ionic/electronic charge separations^60^. Also *CPE*_*DL*_ is used instead of double layer capacitance (*C*_*DL*_) because the semicircles are distorted. Additionally, the high frequency intercept on the real axis represents the series resistance (*R*_*Ω*_), which corresponds to the combination of electrolyte (ionic) resistance, internal resistance of active material and the contact resistance at the solid-liquid interface^61^.

The linear part of the Nyquist plot corresponds to the Warburg impedance (*W*), which is manifested due to the combination of (*a*) diffusion of ions from electrolyte to titania nanostructures, and (*b*) the insertion capacitance due to the accumulation of charges at the electrode surface. Warburg impedance is often represented by Warburg coefficient (*ψ*_*w*_), through which Warburg impedance and diffusion coefficients can be predicted^62^. The shape of this part reveals the capacitive behavior of the electrode, although an angle around 45 ° against the real axis depicts that the electrochemical process is either slightly diffusion controlled (which is also observed by others for TiO_2_ nanoparticles/nanorods/nanofibers^62,63^) and/or occurs as a result of frequency dispersion due to a wide size distribution/roughness variations within the active materials^60^.

According to the shape of the Nyquist plot presented in Fig. 8, the EIS data are fitted with a typical equivalent circuit {*R*_*Ω*_ + *CPE*_*DL*_||(*R*_*ct*_ + *W*)} shown in the inset of Fig. 8(a) ^62,63^. The fitted values are presented in Table 3. The sample prepared at 0.5 M KOH (2D platelet + 1D nanorod mixture sample) showed lowest value of *R*_*ct*_ (0.82 Ω) and highest value of *CPE*_*DL*_ (12.0 μF), against other samples (as well as reported data, *R*_*ct*_ ~ 150 Ω–730 Ω^62,63^), indicating much easier charge transfer rate at the electrode/electrolyte interface, which boost the electronic conductivity, and hence improve the rate capability and cycle life for superior supercapacitor applications. The fitted EIS data are also consistent with the CV and GCD data (Figs 6, 7 and Table 1), where 2D platelet + 1D nanorod mixture sample showed higher specific capacitances against other samples, indicating highly reproducible and stable electrochemical characteristics of the samples.

It is noteworthy in this connection that, for anodized Ti-TiO_2_ nanotube arrays (and similar porous electrodes), several groups used a different equivalent circuit {*R*_*Ω*_ + *C*_*E*_||*R*_*E*_ + *CPE*||(*R*_*ct*_)}^21,64,65^, which is described in Fig. S4 of Supplementary data. In our case, since the samples are not porous in nature, the equivalent circuit shown in the inset of Fig. 8(a) would be more appropriate, which is also used by others for non-porous TiO_2_ nanofibers and nanoparticles^62,63^. But the above circuit (in Fig. S4) involves the direct growth of TiO_2_ on Ti substrate (like the current case), containing a resistive parameter (*R*_*E*_), that corresponds to the charge flow resistance across the Ti/TiO_2_ interface of the electrode, which provides a qualitative measurement of the interfacial resistance across the metal-metal oxide layers. Since the importance of our work is to minimize this interfacial resistance to improve the electrochemical performance, therefore a qualitative comparison of this interfacial resistance of our sample against the previous reports (through the analysis of the equivalent circuit presented in Fig. S4), would be interesting to corroborate our claim that the *in-situ* hydrothermal surface modification of the Ti substrate into titania nanostructures indeed minimizes the Ti-TiO_2_ interfacial resistance to improve the electrochemical properties. The EIS fitted data according to the equivalent circuit shown in Fig. S4 is presented in Table S2. The *R*_*E*_ values for all the samples range between 330 Ω and 750 Ω, which is considerably smaller than the reported values (~1450 Ω–700 kΩ^21,64,65^). This clearly validates the importance of the current fabrication technique to produce interfacial-defect-free Ti-TiO_2_ electrodes for superior electrochemical activities. Additionally, Table S2 further reveals the fact that the 2D platelet + 1D nanorod mixture sample has smaller charge transfer resistance (*R*_*ct*_) against other samples, which is consistent with Table 3, indicating highly stable and reproducible electrochemical properties of our samples. The reason for this superior electrochemical property of the 2D platelet + 1D nanorod mixture sample against others has already been described in the previous section, which is mainly due to the unique morphology which manifests higher active sites (and hence, higher utilization of the active materials) in terms of greater roughness at the 2D platelet structures and higher surface-to-volume ratio in the 1D nanorod structures.

Finally, the energy and power performance of the as-fabricated electrodes are analyzed by the Ragone plot of areal energy density (*ξ*, in Wh cm^−2^) versus power density (P, in W cm^−2^), which were estimated from the following equations using the GCD curves^66^:

8 $$\xi =\frac{C{({\rm{\Delta }}V)}^{2}}{2\times 3600}$$

9 $$P=\frac{E\times 3600}{{\rm{\Delta }}t}$$

where Δ*V* (in V) is the potential drop after a full discharge, C is the areal capacitance (in F cm^−2^) and Δ*t* is the discharge time (in sec), as mentioned above. It is noteworthy in this connection that, although the Ragone plots are generally used for a packaged device, instead of a half cell (or an electrochemical electrode), but the advent of new electrode materials as well as conventional materials with novel nanostructured morphologies, it would be pertinent to isolate the energy/power performance of individual components of the half cell (like electrode or electrolyte) to understand the effects of these components on the overall electrochemical performance while avoiding the cost of developing a packaged device^67^. Therefore, Ragone plots for nanostructured titania electrodes are presented in Fig. 8(c), which depict the superior energy and power performance of the titania electrode prepared at 0.5 M KOH solution (2D platelet + 1D nanorod mixture sample) against the other samples. Evidently, this is consistent with the CV, GCD and EIS data, indicating highly stable and reproducible electrochemical performance of the nanostructured titania electrodes.

## Conclusions

A simple alkali-controlled hydrothermal technique is used to *in-situ* modify Ti substrate surface into titania nanostructures. Depending on the KOH concentration, the substrate surface is reconstructed into either a 2D platelet structure or a 1D nanorod/nanofiber structure or a mixture of both. XRD analysis depicts high crystallinity of all the nanostructures with higher weight fraction of rutile TiO_2_ phase. XPS spectra reveal that all the samples have identical chemical compositions, indicating that the hydrothermal surface modification process is mainly controlling the morphology of the samples without affecting its chemical properties. The hydrothermal process involves the alkali-controlled conversion of metallic Titanium into hydrated titania, followed by simultaneous dissolution and growth of titania nanostructures in aqueous KOH solution. Depending on the concentrations of aq. KOH, a competition between the dissolution and growth process is manifested, and based on the relative dissolution/growth rates, the dimension of the nanostructures are controlled into a 2D platelet structure and/or 1D nanorods/nanofibers structure. Additionally, some amounts of K-polytitanates are also precipitated on the titania nanostructures, which are subsequently converted (partially) into anatase titania under air-annealing. The importance of the process is that the surface of the Ti substrate is directly converted into titania nanostructures via a bottom up technique (instead of the conventional top-down approach), resulting in a near-defect-free Ti-TiO_2_ interface, which profoundly improved the charge transfer capability for highly enhanced electrochemical properties of the as-synthesized nanomaterials in superior supercapacitor applications.

## Experimental procedure

### Materials and Method

The experimental procedure is a two-step process. In the 1^st^ step, a hydrothermal route is adopted to *in-situ* modify the surface of the Ti substrate into titania nanostructures, and in the 2^nd^ step, these surface-modified Ti-TiO_2_ samples are directly used as the electrochemical microelectrode (without any modifications) for supercapacitive characterizations.

The hydrothermal method is a simple, one-pot process, which involves the use of commercially available Ti discs into a hydrothermal chamber containing potassium hydroxides at varied concentrations under elevated temperature and pressure. Precisely, Ti discs (Sigma Aldrich, 99% purity, 14 mm diameter, 4 mm thickness) were first polished with SiC emery papers (water-proof, electro-coated abrasive papers, O-Shung Abrasive, Korea, grit sizes of 800 and 2000) followed by ultrasonic cleaning in acetone (95%, Duskan Pure Chemicals Co. Ltd., Korea) and DI water (resistivity 18.2 MΩ) and drying in a vacuum oven (6030 A, DZF, MTI Korea) at 60 °C for 1 hour. Then these Ti discs were inserted into indigenously built PTFE [poly(tetra-fluoroethylene)] - lined stainless steel autoclaves containing different concentrations (0.25 M, 0.5 M, 1.0 M and 5.0 M) of KOH (95%, Duskan Pure Chemicals Co. Ltd., Korea) and were subjected to hydrothermal treatment (at 250 °C, 10–12 MPa for 5 hrs.) in a box furnace (Lindberg/Blue M, Thermo Scientific, Korea). Thereafter, the samples were collected, rinsed in DI water and dried, followed by air-annealing at 500 °C for 2 hours in the box furnace to improve the crystallinity and to remove various surface states/defects for better electrochemical performance. The details of the hydrothermal process are described elsewhere^5^.

Also a schematic and pictorial diagram is presented in Fig. S1 (Supplementary data), showing various hydrothermal conditions used to get different nanostructured surfaces. The diagram shows that, by varying the KOH concentrations from 0.25 M to 5.0 M, 2D titania nanoplatelets structures to 1D titania nanorods/nanofibers were fabricated on the Ti substrate (more details are described later). Also the right-hand panel describes the relative rates of dissolution, growth and precipitation of metal oxides as a function of KOH concentrations, details of which are explained later.

In the 2^nd^ step, these surface-modified Ti-TiO_2_ samples are directly used in an electrochemical workstation as the working electrode with proper sample holding arrangements so that the titania nanostructures are exposed to the electrolyte as the active material and the back-contacted Ti substrate is used as the current collector. The description of electrochemical characterization is detailed below.

### Characterizations

The surface morphologies of the samples were inspected by field emission scanning electron microscope (FESEM, S-4200, Hitachi, Japan). The elemental compositions of the hybrids were measured using an energy-dispersive X-ray analysis (EDX) instrument attached to the FESEM system. To improve the conductivity of the powdered samples during FESEM measurements, an ultrathin layer of Pt was sputter-coated onto the sample surface (E-1030 Ion Sputter, Hitachi, Japan).

The nanostructures of the samples were examined using a high-resolution transmission electron microscope (HRTEM, Tecnai G2 F20 STWIN, USA) with a 200 kV field emission electron gun in Schottky mode. For HRTEM imaging, small amount of nanostructures were scratched off from the sample surfaces and dispersed in ethanol, followed by sonication for 5 min, drop-casting onto a commercially available carbon-coated copper grid, and dried under a visible lamp for 5 min. The TEM images were analyzed using commercial digital micrograph instrument software (version 1.82.366, Gatan Inc., USA).

The crystallinity of the samples was analyzed on a PANalytical X’Pert PRO X-ray diffractometer using Cu K_*α*_ radiation (0.154056 nm) at 40 kV and 30 mA. The data were collected in the range of 10 to 90° with a step size of 0.02°. The crystallite sizes (*L*) were calculated from the diffraction data using standard Scherrer formula [$L=\frac{k\lambda }{\beta \,\cos \,\theta },$] where *β* is the full-width-at-half-maxima (FWHM, in radian) of the diffraction peaks, *θ* is the corresponding Bragg angle (in degrees) of the diffraction peaks, *λ* is the x-ray wavelength used (=0.154056 nm) and *k* is the shape factor (dimensionless), typically ~0.9].

The surface properties and chemical composition of the prepared samples were investigated by X-ray photoelectron spectroscopy (XPS) analysis on an X-ray photoelectron spectrometer (K-alpha, Thermo Scientific, USA). The samples were excited using monochromatic Al Kα X-ray radiation (1486.6 eV), and the data were recorded and processed using the commercial software Avantage (version 5.932, Thermo Scientific, USA). All experiments were performed with pass energies of 200 and 30 eV and step sizes of 1 and 0.1 eV for the survey and high-resolution spectra, respectively. Binding energies were measured using extrinsic carbon as an internal standard (C 1 s = 284.8 eV). Each core level spectrum was first fitted with a Shirley-type background and then deconvoluted into various components using GL30 [a mixture of Gaussian (70%) and Lorentzian (30%)] in Avantage. Atomic force microscopy (AFM) imaging and surface roughness measurements were carried out using Digital Instruments (Nanoscope IIIa, USA) Scanning Probe Microscope Controller.

### Adhesion Test

Since the self-source Ti surface is hydrothermally reconstructed into 1D/2D titania nanostructures, they naturally adhered to the substrate, which was substantiated by a Scotch tape adhesion test. The qualitative testing was performed as follows: a strip of pressure-sensitive tape was firmly attached manually across the sample surface. The Ti substrate was fixed on a platform by proper arrangements before the tape attachment. Thereafter, within 60.0 s of the attachment, the tape was removed by a rapid pulling force, and was applied approximately perpendicularly to the test area. A visual examination of the tape and the test area of the samples showed no evidence of removal of the nanostructured surface from the substrate, indicating very good adhesion of the nanomaterials.

### Electrochemical Characterizations

Electrochemical experiments were carried out in an electrochemical workstation (CHI 760E, CH Instruments Inc., USA) using a three-electrode configuration at room temperature with 1.0 M KCl solution as the active electrolyte. Ag/AgCl (agar-saturated KCl) is used as the reference electrode, a platinum wire as the counter electrode and the titania nanostructures on the Ti substrate as the working electrode. For the electrochemical performances of the unique nanostructures, the samples were investigated through cyclic voltammetry (CV), galvanostatic charge-discharge cycling (GCD) and potentiostatic impedance spectroscopic (EIS) measurements. The CV was performed over a voltage range of −0.9 to 0.2 V at various scan rates ranging from 1.0 to 100.0 mV s^−1^. The GCD cycling was carried out by varying the charging-discharging time at different current densities ranging from 100.0 to 500.0 μA cm^−2^. EIS measurements were performed between 100.0 kHz and 0.001 Hz using a 5.0 mV *rms* sinusoidal modulation at a bias potential of −0.2 V.

From all the three processes (CD, GCD and EIS), the specific capacitances (*C*_*s*_) for all the samples were calculated. From CV data, the *C*_*s*_ values (F cm^−2^) were calculated by integrating the area under the CV curves based on the Eq. 7. Similarly, from GCD data, the *C*_*s*_ values (F cm^−2^) were calculated according to Eq. 8. Additionally, from the EIS data, various electrochemical parameters (like charge transfer resistance, series resistance, double layer capacitance/constant phase elements etc.) were estimated, using an equivalent circuit configuration of the sample electrodes. All data generated during and/or analyzed during the current study are included in this article (and its Supplementary Data files).

## Electronic supplementary material

Supplementary Data 1
