# Supplementary material for: Improved electrochemical properties of morphology-controlled titania/titanate nanostructures prepared by in-situ hydrothermal surface modification of self-source Ti substrate for high-performance supercapacitors
Source: Sci Rep. 2017 Oct 16;7:13227. doi: 10.1038/s41598-017-11346-2 (PMC5643498; doi:10.1038/s41598-017-11346-2)
Supplement: Supplementary file 1 — Supplementary Data 1 [file 41598_2017_11346_MOESM1_ESM.pdf]

SUPPLEMENTARY DATA

Improved electrochemical properties of morphology-controlled titania/titanate nanostructures prepared by in-situ hydrothermal surface modification of self-source Ti substrate for high-performance supercapacitors

Arghya Narayan Banerjee[1], V C Anitha[2]*, Sang W Joo[1]*

[1]School of Mechanical Engineering, Yeungnam University, Gyeongsan 712-749, Republic of Korea

[2]Center of Materials and Nanotechnologies, Faculty of Chemical Technology, University of Pardubice, Nam. Cs. Legii 565, 53002 Pardubice, Czech Republic

*Corresponding authors:

vc.anitha@gmail.com (VCA);

swjoo@yu.ac.kr (SWJ); Ph. +82-53-810-3239, Fax: +82-53-810-2062

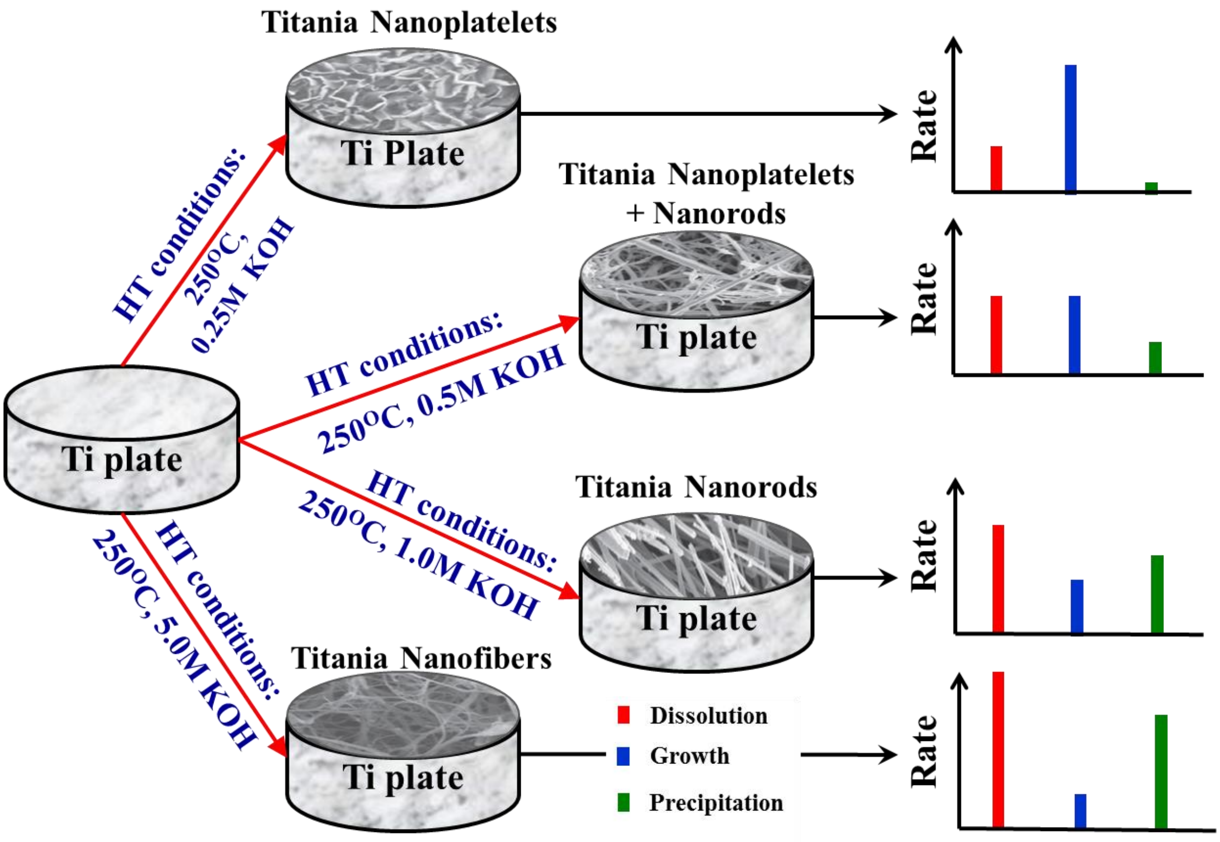

Figure S1: Schematic and pictorial representation of the hydrothermal synthesis of various titania nanostructures on the self-source Ti substrate under different KOH concentrations. Right-hand panel describes the relative dissolution, growth and precipitation rates of TiO_2 on the Ti substrate as a function of KOH concentrations.

Table S1: A comparison of the 2θ-values of different diffraction peaks observed in the XRD data (Fig. 2b) with the 1999 JCPDS-ICDD File. Also d-values of Rutile titania for (110) and (101) planes obtained from Fig. 3 and Fig. S2 are compared with 1999 JCPDS-ICDD File.

**Peak origin**		**$2\theta_{EXPT}$ (deg.)** (From Fig. 2b)	**$2\theta_{JCPDS}$** (1999 JCPDS-ICDD File)	**1999 JCPDS File Card #**
**TiO (110)**		35.6	35.9	12-0754
**Rutile TiO_2**	**(101)**	36.8	36.2	78-1510
	**(202)**	76.6	76.7	
**Anatase TiO_2**	**(200)**	48.0	48.1	84-1286
	**(105)**	53.6	53.9	
	**(220)**	70.7	70.3	
**Ti**		40.1	40.2	05-0682
		63.2	63.0	
		77.5	77.3	
**K-Titanate**		11.6	11.5	74-0275
		24.4	24.6	
		25.5	25.6	
		29.6	29.8	
**Rutile TiO_2** (From TEM micrographs)		**$d_{110}\|_{EXPT}$ = 0.33 nm** (from Fig. 3)	**$d_{110}\|_{JCPDS}$ = 0.324 nm**	78-1510
		**$d_{101}\|_{EXPT}$ = 0.25 nm** (from Fig. S2)	**$d_{101}\|_{JCPDS}$ = 0.248 nm**	

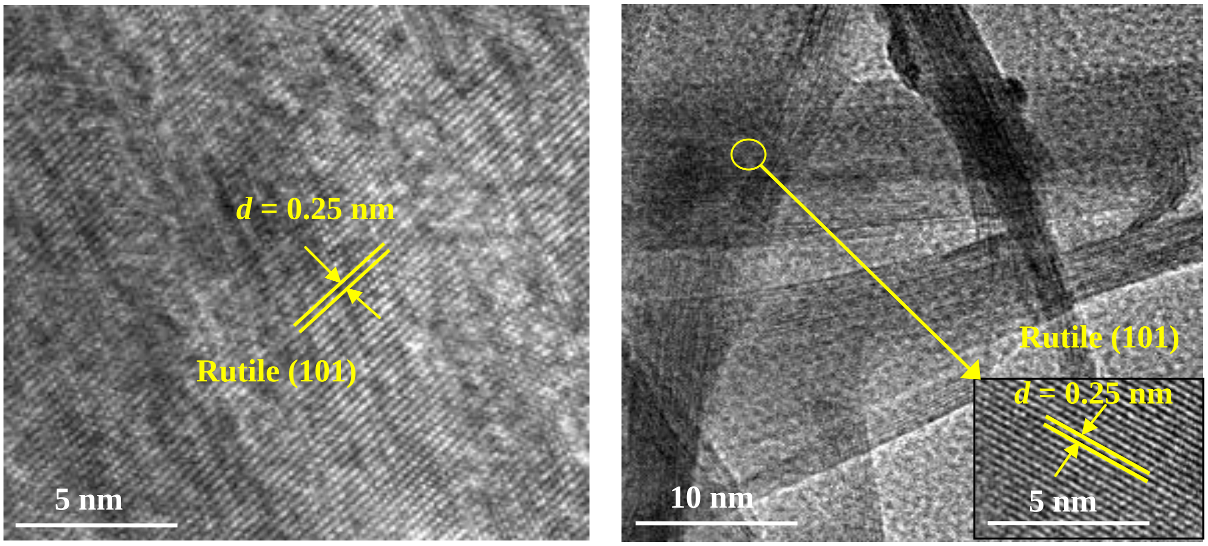

Figure S2: HRTEM micrographs of the interior of (a) titania platelet, (b) titania nanorods. The lattice spacing matches with the Rutile (101) planes with *d*-value around 0.25 nm.

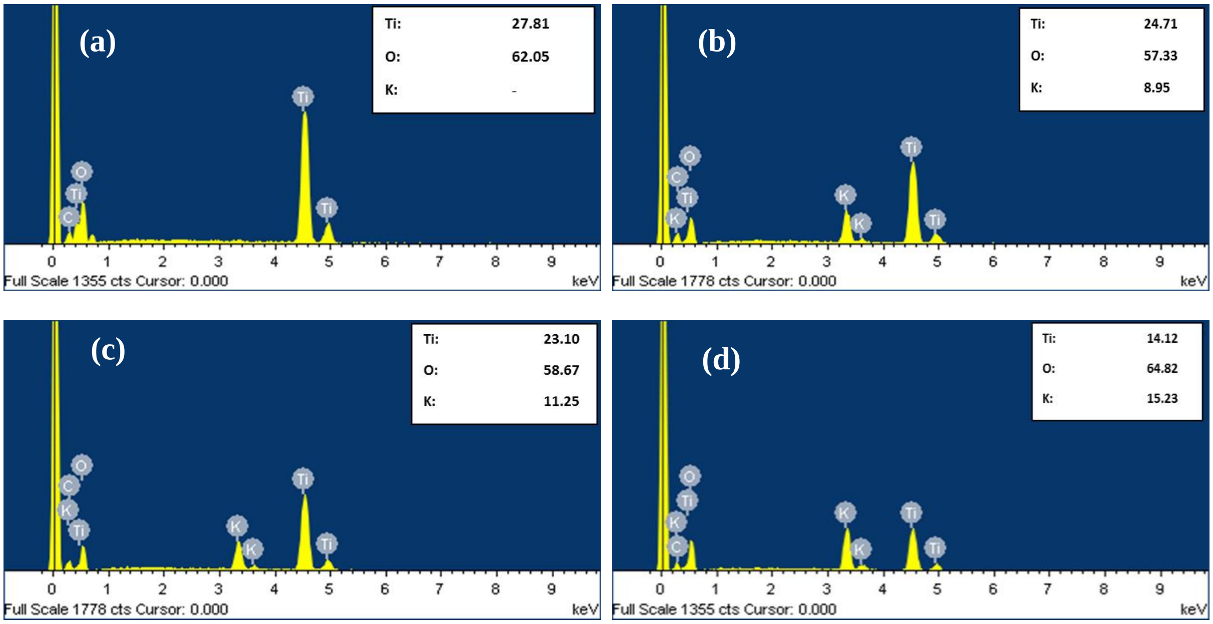

Figure S3: EDX micrograph of titania nanostructures hydrothermally prepared at (a) 0.25 M (2D platelets), (b) 0.5 M (2D platelets + 1D nanorods mixture), (c) 1.0 M (1D nanorods) and (d) 5.0 M KOH solutions (1D nanofibers). Insets represent the corresponding elemental composition.

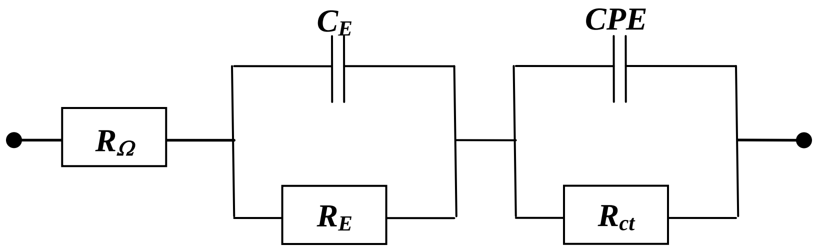

Figure S4: Equivalent circuit representation of Ti-TiO_2 nanostructured electrode. R_Ω: Resistance of the active medium (electrolyte), R_E: Charge flow resistance across Ti-TiO_2 interface, R_{ct}: Charge transfer resistance across TiO_2-Electrolyte interface, C_E: Capacitance of TiO_2, including surface states and Helmholtz double layer, *CPE*: Constant Phase Element defined by *CPE-T* {electrostatic capacitance, (in Farad)} and *CPE-P* {a fitting parameter, α, indicating the degree of deviation from a certain state, (dimensionless)}. For $\alpha = 0, 1, -1$, the *CPE* represents pure resistor, capacitor and inductor, respectively. For $\alpha \approx 0.5$, the CPE corresponds to Warburg impedance, *W* [1-3].

Table S2 EIS fitted data for all the samples according to the equivalent circuit shown in Fig. S4.

Samples	R_Ω (ohm)	C_E (μF)	R_E (ohm)	R_{ct} (ohm)	*CPE* (μF)	α
**0.25M-TO** (*2D platelets*)	2.11	66.5	753.1	9.56	6.99	0.62
**0.5M-TO** (*2D platelets + 1D nanorods*)	2.24	72.6	328.9	7.83	1.45	0.57
**1.0M-TO** (*1D nanorods*)	7.87	96.7	405.3	12.47	2.01	0.53
**5.0M-TO** (*1D nanofibers*)	4.63	208.7	676.6	12.09	2.96	0.50

References

1. M. Salari, S.H. Aboutalebi, A.T. Chidembo, I.P. Nevirkovets, K. Konstantinov and H. K. Liu, *Phys. Chem. Chem. Phys.*, 2012, **14,** 4770–4779.
2. P. Xiao et al. Sensors and Actuators B 134 (2008) 367–372
3. Y. Xie, L. Zhou, J. Lu, J Mater Sci (2009) 44:2907–2915.
